# Is There Really Any Melatonin in Gastric Juice?

**DOI:** 10.1002/hsr2.72160

**Published:** 2026-03-22

**Authors:** David J. Kennaway

**Affiliations:** ^1^ Faculty of Health and Medical Sciences, Adelaide Health and Medical Sciences Building, Adelaide University North Terrace Adelaide South Australia Australia

To the Editor,

The recent paper by Sibande and colleagues [1] aimed to evaluate the relationship between fasting gastric juice melatonin levels and gastroduodenal pathologies. There are a number of problems with this study that make the results unsound. Historically, melatonin was proposed to be synthesized by enterochromaffin cells of the gut based upon the high serotonin content of these cells and the fact that serotonin is a precursor for melatonin. The stomach contains “enterochromaffin‐like” cells that are rich in histamine, not serotonin. So what might be the source of the melatonin? More importantly, the evidence that “concentrations of melatonin in the GIT are much higher than in plasma or the pineal gland” is poor, being based on seriously flawed research [2]. Rather than the rat duodenum having 400 times more melatonin than the pineal gland, it is likely to have more than 20 times less and may well not produce any.

The most troubling issue with this paper is the methodology used to analyze the melatonin content of the gastric juice samples. The authors used ELISA kits from Wuhan Fine Biotech Co. Ltd., China, which use microtiter plates pre‐coated with melatonin. This in itself is an unusual approach since most competitive ELISAs use immobilized antibody on the plates. Melatonin in the sample competes with a fixed amount of melatonin on the solid phase supporter for sites on the biotinylated detection antibody specific to melatonin. The assay requires 50 µL of standards or samples, in this case, 50 µL of highly acidic gastric juice (pH 1.0–2.0).

The authors provided no indication that they attempted to validate the ELISA for gastric juice. They did not attempt to neutralize the acidity of their samples (if any dilution had been performed, the range would have been extended beyond the stated 500 pg/mL). Such low pH levels of the samples would be expected to either release the melatonin bound to the plate or seriously interfere with the antigen‐antibody binding reaction or both, resulting in false high reported values. We can have no confidence that the levels of melatonin reported (approximately 300 pg/mL) are correct or that any of the conclusions can be sustained.

The authors purchased the kits in good faith, but unfortunately, in planning their study, they failed to critically review the literature and lacked an understanding of the need to perform their own validation studies when using the ELISA on an unusual matrix [3].

## Author Contributions

**David J. Kennaway:** writing – review and editing.

## Data Availability

Data sharing is not applicable to this article as no datasets were generated or analyzed during the current study.
